# Non-smooth variational problems and applications

**DOI:** 10.1098/rsta.2021.0364

**Published:** 2022-11-14

**Authors:** Victor A. Kovtunenko, Hiromichi Itou, Alexander M. Khludnev, Evgeny M. Rudoy

**Affiliations:** ^1^ Institute for Mathematics and Scientific Computing, University of Graz, NAWI Graz, Heinrichstr. 36, 8010 Graz, Austria; ^2^ Department of Mathematics, Tokyo University of Science, 1-3 Kagurazaka, Shinjuku-ku, Tokyo 162-8601, Japan; ^3^ Lavrentyev Institute of Hydrodynamics, Siberian Division of the Russian Academy of Sciences, 630090 Novosibirsk, Russia; ^4^ Novosibirsk State University, Department of Mathematics and Mechanics, 630090 Novosibirsk, Russia

**Keywords:** non-smooth variational methods, continuum mechanics

## Abstract

Mathematical methods based on the variational approach are successfully used in a broad range of applications, especially those fields that are oriented on partial differential equations. Our problem area addresses a wide class of nonlinear variational problems described by all kinds of static and evolution equations, inverse and ill-posed problems, non-smooth and non-convex optimization, and optimal control including shape and topology optimization. Within these directions, we focus but are not limited to singular and unilaterally constrained problems arising in mechanics and physics, which are governed by complex systems of generalized variational equations and inequalities. Whereas classical mathematical tools are not applicable here, we aim at a non-standard well-posedness analysis, numerical methods, asymptotic and approximation techniques including homogenization, which are successful within the primal as well as the dual variational formalism. In a broad scope, the theme issue objectives are directed toward advances that are attained in the mathematical theory of non-smooth variational problems, its physical consistency, numerical simulation and application to engineering sciences.

This article is part of the theme issue ‘Non-smooth variational problems and applications’.

During the *8th European Congress of Mathematics (8ECM)* held in June 2021 in Portorož, Slovenia, a mini-symposium on *Non-smooth variational methods for PDEs and applications in mechanics* served as a starting point for collecting potential contributions for the theme issue. Indeed, the congress was postponed from 2020 when we gathered 21 top-quality multi-disciplinary international participants in our mini-symposium, but unfortunately many of them cancelled their participation due to COVID-19 limitations, so we particularly try to recover the intended contributions.

A variational approach is the key word that unites the collection in our theme issue of 13 contributions of the lead authors from 13 countries: Austria, China, Czech Republic, France, Germany, Italy, Japan, Poland, Russia, Spain, Turkey, UK and USA. The new developments in mathematics allow us to treat a variety of non-smooth problems from actual applications in engineering sciences, which cannot be done by conventional methods adopted in the field.

From a mathematical point of view, the developed non-smooth variational methods are based on the Lagrange multiplier approach and dual optimization techniques. The variational problems under consideration are subjected to gradient constraint [1] and unilateral constraints [2,3], they obey non-differentiable objectives and may lose the property of coercivity [4]. These features result in non-smooth optimization, quasi-variational inequalities [5] and hemi-variational inequalities [6]. The cases of stochastic optimal control [7] and coefficient identification from measured data at the boundary [8] belong to the field of inverse problems, which are known to be ill-posed. Well-posedness analysis of the underlying problems provides further investigation with respect to issues of stability and long-time behaviour [9], rate-independent time evolution [10], asymptotic behaviour due to damage [2], variation of geometric and physical parameters, singular perturbations [11], multi-scale analysis and periodic homogenization [12]. The theoretical results are supported by construction of efficient computational techniques, finite and boundary element methods and numerical simulation[7,10].

From the point of view of physics, the variational approach is applied to continuum mechanics of solids as well as incompressible fluids given by the Navier–Stokes [9] and Stokes [6] models. The different types of models for solids describe elastic junctions [2], thermo-elastic composites under mechanical vibration [12], dynamic behaviour of the Euler–Bernoulli beams [8] and thermo-elastic Kirchhoff–Love plates [3]. There are considered bodies that exhibit power-law hardening like the Norton–Hoff and Ramberg–Osgood materials [5], and ideal elasto-plastic behaviour [1]. By this, nonlinear boundary conditions are of the first importance for physically consistent modelling. The theme issue studies the unilateral contact appearing for inclusions subject to delamination with cracks [2,3], non-smooth slip [9], frictional contact [4,13] and degenerating Robin-type transmission conditions for the thin reactive heat-conducting inter-phases [11]. The rate-independent evolutionary systems driven by non-convex energies have been suggested [10], which are successful to model properly jump discontinuities in time during quasi-brittle crack propagation. The elaborated mathematical and mechanical description gives an impact to practise testing methodologies by rigid punch indentation [5], in fracture mechanics and seismology [13].

In this way, the different subject areas outline the topic of the theme issue and its novelty with respect to non-smooth variational problems and their applications.

## Data Availability

This article has no additional data.
